# Development and application of a quadruplex real-time PCR method for Torque teno sus virus 1, Porcine circovirus type 2, pseudorabies virus, and porcine parvovirus

**DOI:** 10.3389/fcimb.2024.1461448

**Published:** 2024-10-16

**Authors:** Fushi Quan, Yulu Geng, Yang Wu, Faming Jiang, Xuemei Li, Changqing Yu

**Affiliations:** ^1^ Department of Laboratory Animals, College of Animal Sciences, Jilin University, Changchun, Jilin, China; ^2^ State Key Laboratory for Animal Disease Control and Prevention, Harbin Veterinary Research Institute, Chinese Academy of Agricultural Sciences, Harbin, China; ^3^ Engineering Center of Agricultural Biosafety Assessment and Biotechnology, School of Advanced Agricultural Sciences, Yibin Vocational and Technical College, Yibin, China

**Keywords:** PMWS, TTSuV 1, PCV2, PRV, PPV, quadruplex, qPCR

## Abstract

**Introduction:**

In clinical diagnosis of porcine diseases, co-infection with multiple viruses often leads to similar clinical symptoms. Postweaning multisystemic wasting syndrome (PMWS) can be caused by infections with TTSuV or PCV2, while PCV2, PRV, and PPV can cause respiratory and reproductive disorders in pigs. The overlapping clinical and pathological features of these infections necessitate the development of a rapid and specific method for differentiating and detecting these four DNA viruses.

**Methods:**

In this study, four pairs of primers and TaqMan probes were designed targeting the conserved sequence of TTSuV, the Rep gene of PCV2, the gE gene of PRV, and the VP2 gene of PPV. After optimizing reaction conditions, including annealing temperature, primer concentration, and probe concentration, a quadruplex real-time PCR method was developed.

**Results:**

This method can specifically detect TTSuV1, PCV2, PRV, and PPV simultaneously, with no cross-reactivity with ASFV, CSFV, PRRSV, PEDV, PSV, and TGEV. The minimum detection limit for each virus was 10 copies/μl, and the inter-assay and intra-assay coefficients of variation ranged from 0.33% to 1.43%. Subsequently, 150 clinical samples were tested to evaluate the practical applicability of this method. The positive rates for TTSuV1, PCV2, PRV, and PPV were 8.6% (13/150), 10.67% (16/150), 14% (21/150), and 11.33% (17/150), respectively.

**Discussion:**

The results indicate that the established quadruplex real-time PCR method can assist in the accurate and rapid diagnosis of TTSuV1, PCV2, PRV, and PPV in clinical settings, providing robust support for the prevention and control of these infections.

## 1 Introduction

With the continuous expansion of pig farming, the occurrence of co-infections by various infectious diseases on farms has significantly increased. Notably, co-infections involving certain viruses result in highly similar clinical symptoms, posing substantial challenges for diagnosis and disease prevention (Oba et al., 2023). Torque teno sus virus (TTSuV) is a non-enveloped circular DNA virus in the Anelloviridae family, genus Iotatorquevirus. TTSuV is widespread in multiple countries and is classified into two genotypes, TTSuV1 and TTSuV2, based on nucleotide sequence difference (Zheng et al., 2018; Li et al., 2020). Studies have found that the infection rate of TTSuV1 in Chinese pig herds is higher than that of TTSuV2 (Zheng et al., 2018). Although TTSuV1 alone does not cause significant symptoms, it is associated with various diseases, including postweaning multisystemic wasting syndrome (PMWS) (Baekbo et al., 2012). TTSuV1 is genetically closely related to the Circoviridae family, and co-infection with TTSuV1 and Porcine circovirus 2 (PCV2) has been reported in PMWS cases (McMenamy et al., 2013). PCV2, a non-enveloped single-stranded DNA virus in the Circoviridae family, genus Circovirus, is the primary pathogen causing PMWS, which manifests as weight loss, respiratory distress, and significant lymph node enlargement in piglets (Yang et al., 2022). PCV2 infection is common in large-scale pig farms worldwide. Both PCV2 and pseudorabies virus (PRV) can cause respiratory and reproductive disorders in pigs (Chen et al., 2022), complicating clinical diagnosis (Zhan et al., 2021; Chen et al., 2023). PRV, a double-stranded linear DNA virus in the Herpesviridae family, can infect pigs of all ages, particularly causing high mortality rates in piglets and reproductive disorders in pregnant sows, causing huge economic losses for the pig industry (Zheng et al., 2022). Despite long-term immunization and antibody detection measures achieving some control over PRV in China, emerging PRV variants present new challenges for PRV prevention and control (Hu et al., 2023). When diagnosing porcine reproductive disorders, porcine parvovirus (PPV) infection should also be considered, as PPV can cause reproductive disorders in pigs (Chen et al., 2023; Li et al., 2024). PPV, a single-stranded negative-sense DNA virus in the Parvoviridae family, can infect sows, leading to abortion, stillbirths, and mummified fetuses in first-litter sows, with the sows themselves showing no obvious symptoms. PPV infection has been found in almost all pig-raising countries (Streck and Truyen, 2020). In summary, when PMWS or porcine reproductive disorders occur, it is crucial to quickly and accurately determine whether co-infection with the four DNA viruses is present to effectively control the outbreak. Therefore, there is a need to establish a method capable of simultaneously and accurately detecting these four DNA viruses.

Currently, several serological detection methods are available for virus detection, including immunofluorescence technology, immunochromatography, and the indirect immunofluorescence assay (Chen et al., 2023; Wu et al., 2023; Chen et al., 2024). However, these techniques are time-consuming and unsuitable for large-scale sample testing. Polymerase chain reaction (PCR), real-time PCR, and enzyme-linked immunosorbent assay (ELISA) have also been reported for detecting these viruses (Cao et al., 2023; Hou et al., 2023). Due to the immature immune system and low antibody levels in response to these four DNA viruses, ELISA is less effective than PCR for their detection. Conventional PCR is less sensitive than probe-based real-time PCR. Probe-based real-time PCR is a technique that utilizes fluorescent probes for virus detection and quantification (Liu et al., 2023). Compared to dye-based PCR and traditional PCR methods, this technique offers superior sensitivity. However, existing probe-based real-time PCR methods cannot simultaneously detect TTSuV1, PCV2, PRV, and PPV. Thus, establishing a quadruplex probe-based real-time PCR detection method is crucial.

In this study, four specific primer pairs and four specific probes were designed based on the highly conserved sequences of these four DNA viruses (e.g., the Rep gene of PCV2, the gE gene of PRV, and the VP2 gene of PPV) (Peng et al., 2016; Zhao Y. et al., 2020). Reaction time and temperature were optimized, and sensitivity, repeatability, and specificity were evaluated. The results indicate that the established quadruplex TaqMan real-time fluorescent quantitative detection method for TTSuV1, PCV2, PRV, and PPV is faster and more accurate. This method holds practical value for the clinical diagnosis and prevention of TTSuV1, PCV2, PRV, and PPV, providing a rapid and accurate diagnostic tool for epidemiological investigations and veterinary clinical diagnostics.

## 2 Materials and methods

### 2.1 Primer and probe design

All available sequences of the TTSuV1 gene, PCV2 Rep gene, PRV gE gene, and PPV VP2 gene from GenBank (as of March 1, 2024) were aligned multiple times. Using MEGA7 software, we identified the highly conserved regions of TTSuV1, PCV2, PRV, and PPV. Four pairs of primers and four corresponding probes were then designed using Oligo (Version 7.60) software. To prevent interference among fluorescent signals in this multiplex system, four fluorophores with significantly different wavelengths were chosen for the probes: FAM for TTSuV1, NED for PCV2, CY5 for PRV, and VIC for PPV. The sequences of the primers and probes are shown in **Table 1**. These primers and hydrolysis probes were synthesized by Sangon Biotech (Shanghai) Co., Ltd.

**Table 1:** Primers and probes of TTSuV1, PCV2, PRV, and PPV.

Primer/Probe Name	Sequence 5’-3’	Gene	Length(bp)
TTSuV1-F	TGGTACTCCTCAACTGCTGTC		168
TTSuV1-R	CTTCCTCCGTGGATTGTTCTG		
TTSuV1-Probe	FAM-CTTCCTCCGTGGATTGTTCTG-MGB		
PCV2-F	TGGTACTCCTCAACTGCTGTC	Rep	218
PCV2-R	CTTCCTCCGTGGATTGTTCTG		
PCV2-Probe	NED-CTTCCTCCGTGGATTGTTCTG-MGB		
PRV-gE-F	TTCCACTCGCAGCTCTTCT	gE	156
PRV-gE -R	GAGTCGCCCATGTCCGAGA		
PRV-gE -Probe	Cy5-ACACGTTCGACCTGATG-MGB		
PPV-F	GCAAGCTTAATGGTCGCACTAG	VP2	176
PPV -R	GTTTCACTTCTAGGTGCTGCTG		
PPV -Probe	VIC-ACCAATAACACACTTCCA-MGB		

### 2.2 Viruses, nucleic acids, and clinical samples

Nucleic acid samples for TTSuV1, PCV2, PRV, and PPV were obtained from our laboratory’s repository. Positive nucleic acids for ASFV, CSFV, and PRRSV were generously provided by Dr. Qiang Zhang from Huazhong Agricultural University. These positive nucleic acids were used for plasmid standard construction and specificity tests. From 2021 to 2023, we collected a total of 150 clinical samples, including lymph nodes, blood, and anal swabs, from pigs exhibiting respiratory and/or reproductive issues along with progressive weight loss. These samples were collected from farms in Heilongjiang, Jilin, and Shandong provinces and stored in the laboratory for further analysis.

### 2.3 Nucleic acid extraction and reverse transcription

Clinical samples used for specificity assays were processed using a DNA/RNA virus nucleic acid extraction kit (TIANGEN) following the manufacturer’s instructions. The extracted nucleic acids were then reverse-transcribed using the HiScript III RT SuperMix for qPCR (+gDNA wiper) kit (Vazyme Biotech Co., Ltd., Nanjing).

### 2.4 Optimization of the quadruplex real-time quantitative PCR assay

To optimize the qPCR detection parameters, including annealing temperature and the concentrations of primers and probes, a single-variable control method was employed. The PCR reaction was carried out in a total volume of 20 μL, which included 2× Animal Detection U+ Probe qPCR Super Premix (Vazyme Biotech, China), 2 μL of template, primers (0.2–1.0 μM), and distilled water to make up the final volume. All reactions were amplified using the Applied Biosystems QuantStudio 5 (Thermo Fisher Scientific). The amplification protocol was as follows: initial incubation at 37°C for 2 minutes, initial denaturation at 95°C for 30 seconds, followed by 40 cycles of denaturation at 95°C for 10 seconds, annealing at specified temperatures (56°C, 57°C, 58°C, 59°C, 60°C) for 30 seconds, and extension. Fluorescence signals were analyzed at the end of each cycle.

### 2.5 Construction of standard plasmids and establishment of standard curves

DNA templates of TTSuV1, PCV2, PRV, and PPV preserved in the laboratory were used to amplify target segments via PCR. The primers used for amplification were identical to those in the four-channel fluorescence quantitative PCR method. PCR fragments were cloned into the pMD18-T vector (Takara Biomedical Technology (Beijing) Co., Ltd) using TA cloning and confirmed by DNA sequencing. Plasmid DNA containing the PCR inserts was extracted using the Omega EZNA Plasmid Mini Kit I and validated by DNA sequencing for accuracy. Standard plasmids, pMD-TTSuV1, pMD-PCV2, pMD-PRV, and pMD-PPV, were quantified using a NanoDrop spectrophotometer (Thermo Fisher, Waltham, MA, USA). Plasmid copy numbers were calculated, mixed in equal volumes, and diluted from 1×10^9 copies/μL to 1×10^2 copies/μL for use as templates in multiplex real-time fluorescence quantitative PCR to generate standard curves. Efficiency (E value), correlation coefficient (R^2), and standard equations were calculated.

Plasmid copies number/μL = (6.02×10^23)×(X* ng/μL (10^-9)/construct plasmid length (bp)×660 (Wang et al., 2023)

*X: Concentration of recombinant plasmid

### 2.6 Specificity of multiplex real-time fluorescence quantitative PCR

DNA/cDNA of ASFV, CSFV, PRRSV, PEDV, PSV, and TGEV were used as templates to construct the multiplex real-time PCR to verify the specificity of the method. Additionally, DNA of TTSuV1, PCV2, PRV, and PPV served as positive controls, while distilled water was used as a negative control.

### 2.7 Sensitivity of multiplex real-time fluorescence quantitative PCR

To evaluate the limit of detection, the pMD-TTSuV1, pMD-PCV2, pMD-PRV, and pMD-PPV standard plasmids were mixed and diluted 10-fold from 1×10^9 copies/μL to 1×10^1 copies/μL under optimized conditions for use as templates.

### 2.8 Repeatability analysis

To assess the repeatability of the multiplex real-time PCR assay, three gradients of positive plasmid templates containing 1×10^7 copies/μL, 1×10^5 copies/μL, and 1×10^3 copies/μL were mixed in equal volumes and used as templates. The established multiplex PCR assay was performed for detection. Both intra-assay and inter-assay experiments were conducted three times each, with a two-week interval between experiments. Coefficients of variation (CVs) from intra-assay and inter-assay measurements were calculated to evaluate the repeatability of the assay.

### 2.9 Clinical sample testing

In the final stage, our laboratory collected a total of 150 clinical samples including lymph nodes, blood, and rectal swabs from pigs showing respiratory and/or reproductive problems along with progressive weight loss. These samples were subjected to detection using the multiplex qRT-PCR method developed in this study to analyze all cDNA from clinical specimens. To evaluate the reliability of the quadruple fluorescent quantitative PCR results, the clinical samples were also validated using conventional singleplex PCR. The concordance rates between the two detection methods were compared and further analyzed.

## 3 Results

### 3.1 Optimization of the quadruplex real-time quantitative PCR assay

The annealing temperature optimization was conducted within the range of 54°C, 56°C, 58°C, and 60°C. The optimal annealing temperature was determined to be 56°C. When the concentrations of primers for TTSuV1, PCV2, PRV, and PPV were set at 0.1, 0.2, 0.15, and 0.1 μM respectively, and probe concentrations were 0.05, 0.1, 0.05, and 0.2 μM, the amplification curves in all four fluorescence channels showed the highest peaks with lower Ct values. The Ct values for the negative controls showed either no fluorescence signal or Ct values greater than 35. Therefore, a Ct value less than 35 was defined as positive. Samples with Ct values between 35 and 40 were considered borderline and required re-sampling.

### 3.2 Standard curve creation

**Figure 1:**
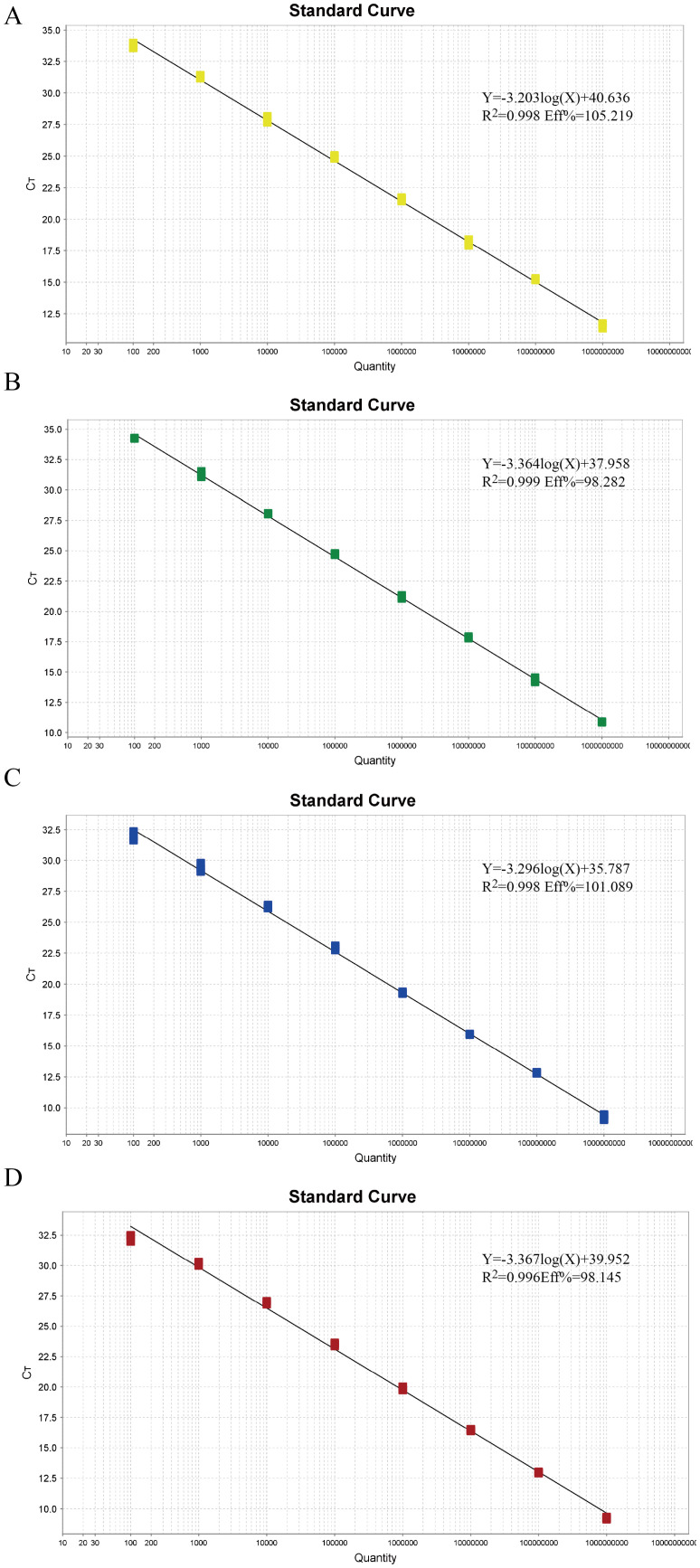
The standard curves of the quadruplex real-time quantitative PCR assay. **(A–D)** Standard curves of the standard plasmid pMD-TTSuV1 **(A)**, pMD-PCV2 **(B)**, pMD-PRV **(C)**, and pMD-PPV **(D)** at final reaction concentrations ranging from 1.0 × 10^9 to 1.0 × 10^2 copies/µL.

To establish the standard curve, recombinant plasmids were serially diluted 10-fold and mixed in equal volumes, ranging from 1×10^9 copies/µL to 1×10^2 copies/µL. Each gradient of plasmid standard was subjected to multiplex real-time fluorescence quantitative PCR. The amplification efficiencies and correlation coefficients were excellent, with R^2 values of 0.998, 0.999, 0.998, and 0.996 for TTSuV1, PCV2, PRV, and PPV, respectively. The efficiencies (Eff%) were calculated as 105.219%, 98.282%, 101.089%, and 98.145%, respectively. The linear equations were as follows: TTSuV1: Y=-3.203log(X)+40.636, PCV2: Y=-3.364log(X)+37.958, PRV: Y=-3.296log(X)+35.787, and PPV: Y=-3.367log(X)+39.952 (**Figure 1**).

### 3.3 Specificity of multiplex qPCR detection

**Figure 2:**
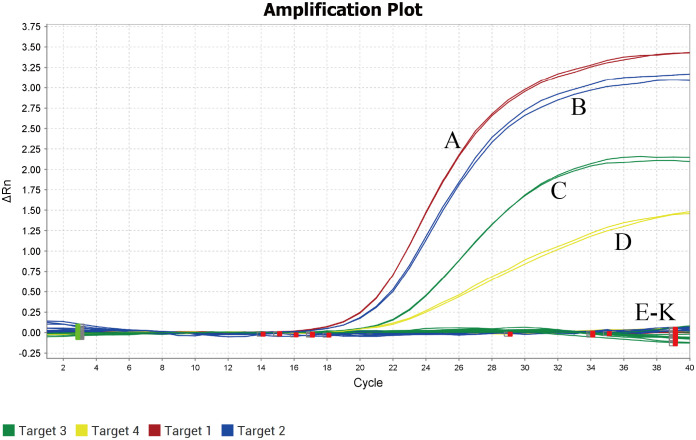
Specificity analysis of the quadruplex real-time quantitative PCR assay. **(A)** TTSuV1; **(B)** PCV2; **(C)** PRV; **(D)** PPV; **(E-K)** ASFV,CSFV, PRRSV, PEDV, PSV and TGEV.

DNA from TTSuV1, PCV2, PRV, and PPV served as positive controls, while ASFV, CSFV, PRRSV, PEDV, PSV, and TGEV DNA/cDNA were used as templates, and ddH2O as a negative control. TaqMan qPCR was conducted under optimal conditions identified in fluorescence quantitative PCR. The results demonstrated amplification of TTSuV1, PCV2, PRV, and PPV nucleic acids only in the positive samples (**Figure 2**), with no amplification observed for other viral pathogens. These findings indicate that the method exhibits excellent specificity.

### 3.4 Sensitivity and repeatability analysis

**Figure 3:**
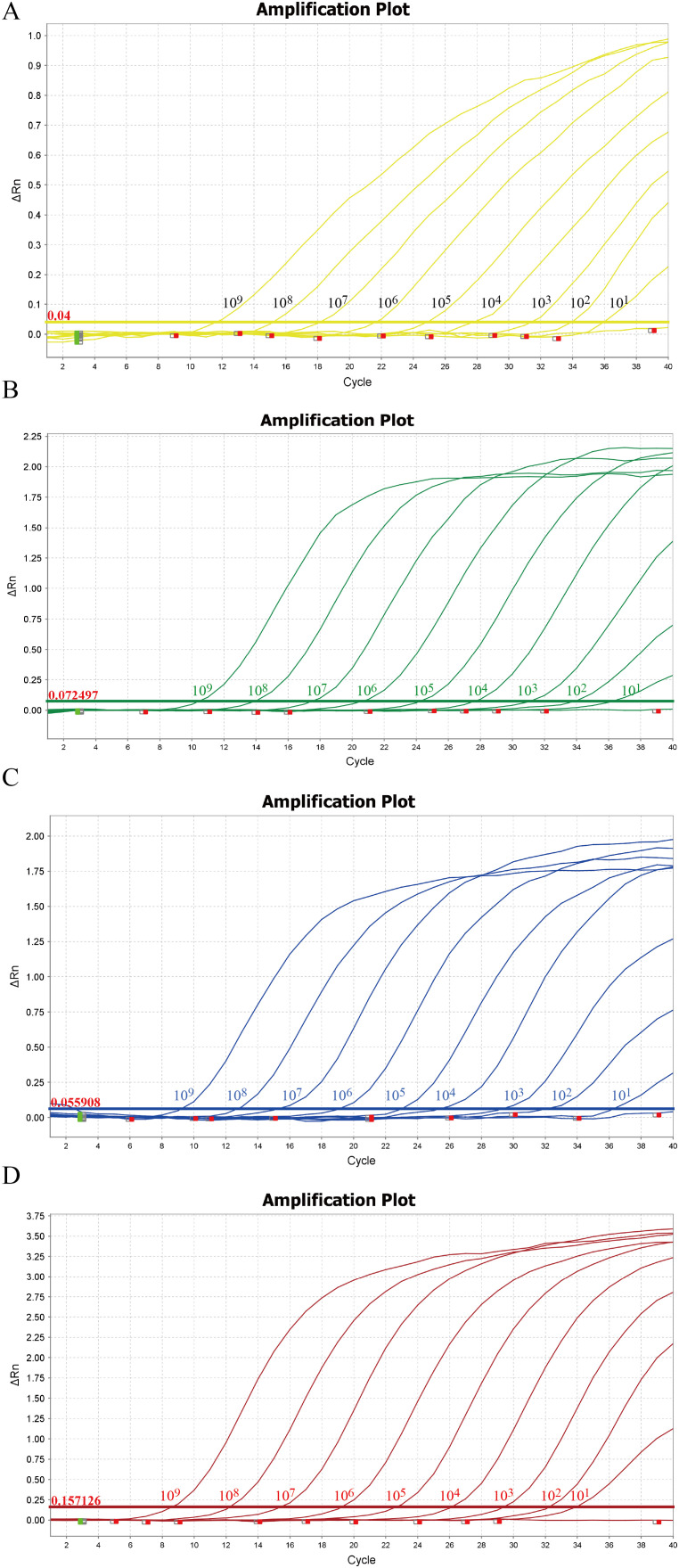
Sensitivity of the quadruplex real-time quantitative PCR assay. The amplification curves were generated by using the standard plasmid pMD-TTSuV1 **(A)**, pMD-PCV2 **(B)**, pMD-PRV **(C)**, and pMD-PPV **(D)**. 1–9: 1.0 × 10^9–1.0 × 10^1 copies/µL (final concentration).

Within the concentration range of 1×10^9 copies/µL to 1×10^1 copies/µL, the sensitivity of the method was evaluated using the optimal reaction conditions for TTSuV1, PCV2, PRV, and PPV. As shown in **Figure 3**, the method exhibited a detection limit of 10 copies/µL for TTSuV1, PCV2, PRV, and PPV, indicating excellent sensitivity. The cutoff Ct value for TTSuV1 and PCV2 positivity was 37, where samples with Ct values ≤37 were considered positive and those >37 were negative. For PRV and PPV, the cutoff Ct value was 35, with samples >35 considered negative.

### 3.5 Repeatability of the quadruplex real-time quantitative PCR assay

The repeatability of the developed multiplex TaqMan qPCR method was evaluated using recombinant standard plasmids at concentrations of 10^7 copies/µL, 10^5 copies/µL, and 10^3 copies/µL as templates. As shown in **Table 2**, the coefficient of variation (CVs) for Ct values ranged from 0.33% to 1.43% in both intra-group and inter-group replicates, indicating excellent repeatability of the method.

**Table 2:** Repeatability of the quadruplex real-time quantitative PCR assay.

Standard plasmid	Concentration of template (copies/μL)	Intra-coefficient of variation		Inter-coefficient of variation	
		X ± SD	CV (%)	X ± SD	CV (%)
pMD- TTSuV1	10^7^	18.215 ± 0.052	0.28	18.302 ± 0.026	0.14
	10^5^	24.168 ± 0.120	0.50	24.621 ± 0.106	0.43
	10^3^	31.027 ± 0.251	0.81	31.105 ± 0.351	1.13
pMD- PCV2	10^7^	14.315 ± 0.162	1.13	14.410 ± 0.101	0.70
	10^5^	21.138 ± 0.111	0.53	21.056 ± 0.245	1.16
	10^3^	27.866 ± 0.316	1.13	27.541 ± 0.277	1.01
pMD- PRV	10^7^	12.715 ± 0.182	1.43	12.605 ± 0.113	0.90
	10^5^	19.308 ± 0.189	0.98	19.425 ± 0.203	0.43
	10^3^	25.899 ± 0.213	0.82	26.014 ± 0.104	0.40
pMD-PPV	10^7^	16.383 ± 0.122	0.74	16.162 ± 0.053	0.33
	10^5^	23.107 ± 0.108	0.47	23.216 ± 0.123	0.53
	10^3^	29.851 ± 0.253	0.85	29.546 ± 0.364	1.23

### 3.6 Clinical sample detection

**Figure 4:**
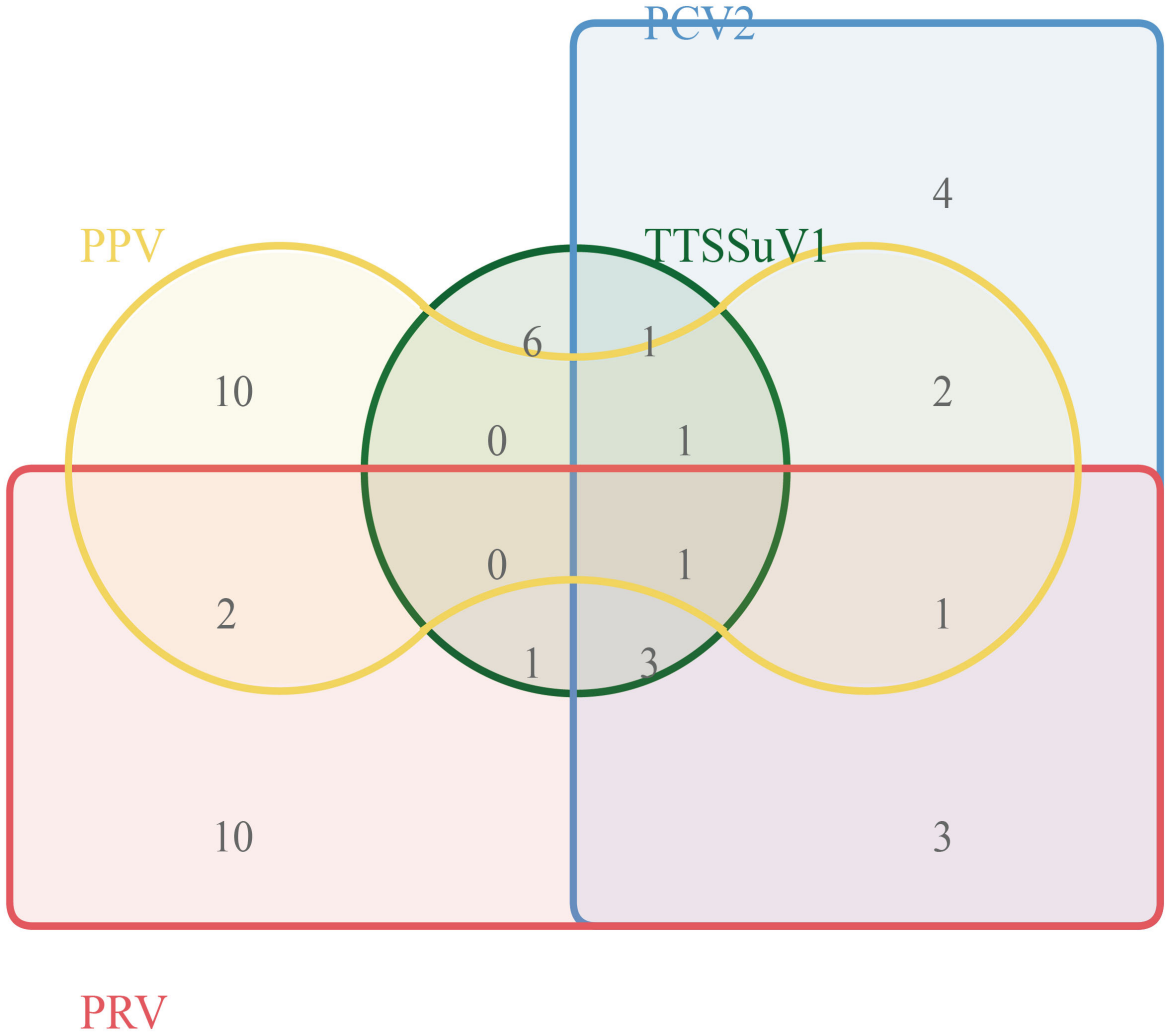
Clinical sample testing.

To further validate the developed method’s clinical applicability in the differential diagnosis of viral pathogens, researchers simultaneously tested clinical samples from 150 pigs presenting with diarrhea symptoms using both industry-standard methods and the multiplex qRT-PCR method developed in this study. The results are summarized in **Figure 4**. According to industry standards, the infection rates of TTSuV1, PCV2, PRV, and PPV were 8.6% (13/150), 10.67% (16/150), 14% (21/150), and 11.33% (17/150), respectively. The co-infection rates of TTSuV1+PCV2, PCV2+PRV, PRV+PPV, TTSuV1+PRV, PPV+PCV2, TTSuV1+PCV2+PRV, PCV2+PRV+PPV, TTSuV1+PCV2+PPV, and TTSuV1+PCV2+PRV+PPV were 0.67% (1/150), 2% (3/150), 1.33% (2/150), 0.67% (1/150), 1.33% (2/150), 2% (3/150), 0.67% (1/150), 0.67% (1/150), and 0.67% (1/150), respectively. These findings underscore the importance of establishing a more sensitive diagnostic method, crucial for timely disease prevention and control.

## 4 Discussion

In the swine industry, mixed infections of infectious diseases represent one of the most serious challenges. When an outbreak occurs on a pig farm, it is rarely caused by a single pathogen. Veterinary professionals must consider the possibility of multiple pathogenic microorganisms causing a single disease manifestation in pigs. For instance, PMWS, commonly observed during farming (Allan et al., 2004), may result from co-infections involving TTSuV1 and PCV2 viruses, among others (Ramos et al., 2018). Therefore, diagnosing the etiology of PMWS requires concurrent testing for TTSuV1 and PCV2 at a minimum (Vlasakova et al., 2014). PCV2 not only causes PMWS but also respiratory and reproductive disorders in pigs (Ouyang et al., 2019). When diagnosing reproductive disorders, potential co-infections with pathogens like PRV, PPV, or PRRSV must also be considered (Chen et al., 2023). Thus, developing a method capable of simultaneously detecting multiple pathogenic microorganisms is crucial for clinical diagnosis of swine diseases.

Conventional PCR and real-time qPCR methods are used for rapid and accurate pathogen detection in clinical settings. Currently, there are reports on detection methods for TTSuV1 targeting the IgG antibody (Gimenez-Lirola et al., 2014). Previous reports have described single fluorescence qPCR methods for detecting TTSuV1, with a reported minimum detection limit of 5×10^2copies/μL (Teixeira et al., 2015). In contrast, the multiplex qPCR method developed in this study achieved a minimum detection limit of 1×10^2 copies/μL for TTSuV1, demonstrating superiority. Similarly, for PCV2, previously reported qPCR methods have a minimum detection limit of 1×10^3 copies/μL (Wang et al., 2020; Chen et al., 2023), whereas the method developed in this study achieved 1×10^2 copies/μL, also an improvement. Comparable improvements were noted for PRV and PPV detection limits (Lyu et al., 2023). While dual fluorescence qPCR methods for PPV4 and PPV6 have been reported, capable of detecting positivity rates ranging from 11.36% to 12.5% for PPV4 and 30.68% to 37.5% for PPV6 in samples from Fujian Province in 2022, no PCR method currently exists for simultaneously detecting TTSuV1, PCV2, PRV, and PPV (Lyu et al., 2023). In 2022, it was reported that fluorescence quantitative detection methods for ASFV, PCV2, and PRV were established. The sensitivity of the PCV2 and PRV detection methods developed in this study is superior to those reported previously (Liu et al., 2023). As these viruses are all DNA viruses, constructing a multiplex qPCR method eliminates the need for nucleic acid reverse transcription, offering advantages in one-step qPCR construction.

In this study, specificity primers and probes were designed based on conserved sequences identified through alignment of GenBank-logged TTSuV1 whole genome sequences, PCV2 Rep gene sequences, and PRV gE gene sequences (Liu et al., 2023). The seven subtypes of PPV, including VP1 to VP7, were evaluated, with VP2 of PPV1 identified as a virulence determinant potentially influencing virus pathogenicity (Zhao K. et al., 2020; Kim et al., 2021). Optimization of reaction time and temperature in the multiplex qPCR method further evaluated sensitivity, repeatability, and specificity. The method utilized four signals (FAM, NED, CY5, and VIC) to detect and distinguish the four target pathogens, showing no wavelength interference and allowing simultaneous fluorescence signal detection in the same reaction tube. Sensitivity tests confirmed detection of fewer than 10 copies of the target genes in standard plasmids containing these four genes, with strong linear correlation between Ct values and standard copy numbers. The method demonstrated specificity in detecting PCV2, PCV3, PPV, and PRV without cross-reactivity with other swine RNA viruses such as CSFV, PRRSV, and PEDV. Furthermore, experimental validation using confirmed ASFV positive samples confirmed the method’s specificity in detecting the four viruses studied in this research (data not shown).

Simultaneously, further testing of additional pathogen-positive samples is needed to validate the broad utility of the developed multiplex real-time PCR method and to conduct further research. Clinical samples from 150 cases were tested to verify the practicality and effectiveness of the method in clinical specimens. The results showed infection rates of TTSuV1, PCV2, PRV, and PPV were 8.6% (13/150), 10.67% (16/150), 14% (21/150), and 11.33% (17/150), respectively. This indicates that PCV2, PPV, and PRV remain prevalent in Hunan Province. Additionally, simultaneous co-infections of PCV2, PCV3, PPV, and PRV with two or more pathogens are common, which may exacerbate immune suppression and inflammatory responses, thereby increasing the likelihood of secondary infections by other pathogens and further exacerbating these diseases. Clinical sample testing revealed that 43 samples (13.65%) still had co-infections of PCV2, PCV3, PPV, and PRV. At the time of the initial design of this study, we consulted the literature and found few reports on the simultaneous infection of these four DNA viruses. Interestingly, through the detection of 150 clinical samples, we discovered a mixed infection of these four DNA viruses in one sample, which drew our attention. The mixed infection of these four DNA viruses is likely to become a new threat to the development of the pig industry and is worthy of focused attention. In the future, we hope that the continuous promotion of our detection technology will provide new technical support for the clinical detection of these four DNA viruses.

In summary, we have developed a multiplex real-time fluorescence quantitative PCR method for the simultaneous identification and detection of TTSuV1, PCV2, PRV, and PPV. This technique enables rapid and precise detection of these viruses in clinical samples, providing a more efficient tool for accurate diagnosis and epidemiological investigation of these viral infections.

## Data Availability

The original contributions presented in the study are included in the article/supplementary material. Further inquiries can be directed to the corresponding authors.

## References

[B1] Allan G. M. McNeilly F. Ellis J. Krakowka S. Botner A. McCullough K.. (2004). PMWS: experimental model and co-infections. Vet. Microbiol. 98, 165–168. doi: 10.1016/j.vetmic.2003.10.009 14741129

[B2] Baekbo P. Kristensen C. S. Larsen L. E. (2012). Porcine circovirus diseases: a review of PMWS. Transbound Emerg. Dis. 59 Suppl 1, 60–67. doi: 10.1111/j.1865-1682.2011.01288.x 22252114

[B3] Cao L. Kong X. Li X. Suo X. Duan Y. Yuan C.. (2023). A customized novel blocking ELISA for detection of bat-origin swine acute diarrhea syndrome coronavirus infection. Microbiol. Spectr. 11, e0393022. doi: 10.1128/spectrum.03930-22 37272819 PMC10434073

[B4] Chen S. Li X. Zhang X. Niu G. Yang L. Ji W.. (2022). PCV2 and PRV coinfection induces endoplasmic reticulum stress via PERK-eIF2alpha-ATF4-CHOP and IRE1-XBP1-EDEM pathways. Int. J. Mol. Sci. 23. doi: 10.3390/ijms23094479 PMC9101680 35562870

[B5] Chen Y. He Z. Luo Y. Su Q. Wang Q. Wang J.. (2024). Tris stabilized AuNPs based lateral flow immunochromatography for the simultaneous detection of porcine epidemic diarrhea virus and rotavirus on-site. . Spectrochim Acta A Mol. Biomol Spectrosc 320, 124670. doi: 10.1016/j.saa.2024.124670 38908108

[B6] Chen Y. Luo S. Tan J. Zhang L. Qiu S. Hao Z.. (2023). Establishment and application of multiplex real-time PCR for simultaneous detection of four viruses associated with porcine reproductive failure. Front. Microbiol. 14, 1092273. doi: 10.3389/fmicb.2023.1092273 36846754 PMC9949525

[B7] Gimenez-Lirola L. G. Gerber P. F. Rowland R. R. Halbur P. G. Huang Y. W. Meng X. J.. (2014). Development and validation of a 4-plex antibody assay for simultaneous detection of IgG antibodies against Torque teno sus virus 1 (TTSuV1), TTSuV2, and porcine reproductive and respiratory syndrome virus types 1 and 2. Res. Vet. Sci. 96, 543–550. doi: 10.1016/j.rvsc.2014.02.014 24650623

[B8] Hou W. Fan M. Zhu Z. Li X. (2023). Establishment and application of a triplex real-time RT-PCR assay for differentiation of PEDV, poRV, and PDCoV. Viruses 15. doi: 10.3390/v15061238 PMC10301752 37376539

[B9] Hu X. Feng S. Shi K. Shi Y. Yin Y. Long F.. (2023). Development of a quadruplex real-time quantitative RT-PCR for detection and differentiation of PHEV, PRV, CSFV, and JEV. Front. Vet. Sci. 10, 1276505. doi: 10.3389/fvets.2023.1276505 38026635 PMC10643766

[B10] Kim S. C. Jeong C. G. Nazki S. Lee S. I. Baek Y. C. Jung Y. J.. (2021). Evaluation of a multiplex PCR method for the detection of porcine parvovirus types 1 through 7 using various field samples. PloS One 16, e0245699. doi: 10.1371/journal.pone.0245699 33508002 PMC7842984

[B11] Li W. Li Y. Li M. Zhang H. Feng Z. Xu H.. (2024). Development and application of a blocking ELISA based on a N protein monoclonal antibody for the antibody detection against porcine reproductive and respiratory syndrome virus 2. Int. J. Biol. Macromol 269, 131842. doi: 10.1016/j.ijbiomac.2024.131842 38679249

[B12] Li G. Wang R. Cai Y. Zhang J. Zhao W. Gao Q.. (2020). Epidemiology and evolutionary analysis of Torque teno sus virus. Vet. Microbiol. 244, 108668. doi: 10.1016/j.vetmic.2020.108668 32402339

[B13] Liu H. Zou J. Liu R. Chen J. Li X. Zheng H.. (2023). Development of a taqMan-probe-based multiplex real-time PCR for the simultaneous detection of african swine fever virus, porcine circovirus 2, and pseudorabies virus in east China from 2020 to 2022. Vet. Sci. 10. doi: 10.3390/vetsci10020106 PMC9964870 36851410

[B14] Lyu Z. Zhang X. Xue S. Yang X. Liu J. Fan K.. (2023). Detection and genetic evolution analysis of porcine parvovirus type 7 (PPV7) in Fujian Province. Infect. Genet. Evol. 115, 105515. doi: 10.1016/j.meegid.2023.105515 37866684

[B15] McMenamy M. J. McKillen J. McNair I. Duffy C. Blomstrom A. L. Charreyre C.. (2013). Detection of a porcine boca-like virus in combination with porcine circovirus type 2 genotypes and Torque teno sus virus in pigs from postweaning multisystemic wasting syndrome (PMWS)-affected and non-PMWS-affected farms in archival samples from Great Britain. Vet. Microbiol. 164, 293–298. doi: 10.1016/j.vetmic.2013.03.009 23578709

[B16] Oba P. Wieland B. Mwiine F. N. Erume J. Dione M. M. (2023). Co-infections of respiratory pathogens and gastrointestinal parasites in smallholder pig production systems in Uganda. Parasitol. Res. 122, 953–962. doi: 10.1007/s00436-023-07797-4 36810670 PMC10006049

[B17] Ouyang T. Zhang X. Liu X. Ren L. (2019). Co-infection of swine with porcine circovirus type 2 and other swine viruses. Viruses 11. doi: 10.3390/v11020185 PMC6410029 30795620

[B18] Peng Z. Ma T. Pang D. Su D. Chen F. Chen X.. (2016). Expression, purification and antibody preparation of PCV2 Rep and ORF3 proteins. Int. J. Biol. Macromol 86, 277–281. doi: 10.1016/j.ijbiomac.2016.01.073 26812108

[B19] Ramos N. Mirazo S. Botto G. Teixeira T. F. Cibulski S. P. Castro G.. (2018). High frequency and extensive genetic heterogeneity of TTSuV1 and TTSuVk2a in PCV2- infected and non-infected domestic pigs and wild boars from Uruguay. Vet. Microbiol. 224, 78–87. doi: 10.1016/j.vetmic.2018.08.029 30269794

[B20] Streck A. F. Truyen U. (2020). Porcine parvovirus. Curr. Issues Mol. Biol. 37, 33–46. doi: 10.21775/cimb.037.033 31822635

[B21] Teixeira T. F. Cibulski S. P. dos Santos H. F. Wendlant A. de Sales Lima F. E. Schmidt C.. (2015). Torque teno sus virus 1 (TTSuV1) and 2 (TTSuV2) viral loads in serum of postweaning multisystemic wasting syndrome (PMWS)-affected and healthy pigs in Brazil. Res. Vet. Sci. 101, 38–41. doi: 10.1016/j.rvsc.2015.05.016 26267087

[B22] Vlasakova M. Leskova V. Sliz I. Jackova A. Vilcek S. (2014). The presence of six potentially pathogenic viruses in pigs suffering from post-weaning multisystemic wasting syndrome. BMC Vet. Res. 10, 221. doi: 10.1186/s12917-014-0221-8 25266874 PMC4194362

[B23] Wang Y. Noll L. Porter E. Stoy C. Dong J. Anderson J.. (2020). Development of a differential multiplex real-time PCR assay for porcine circovirus type 2 (PCV2) genotypes PCV2a, PCV2b and PCV2d. J. Virol. Methods 286, 113971. doi: 10.1016/j.jviromet.2020.113971 32926893 PMC7486290

[B24] Wang H. Xin L. Wu Y. Liu Y. Yao W. Zhang H.. (2023). Construction of a one-step multiplex real-time PCR assay for the detection of serogroups A, B, and E of Pasteurella multocida associated with bovine pasteurellosis. Front. Vet. Sci. 10, 1193162. doi: 10.3389/fvets.2023.1193162 37448584 PMC10336434

[B25] Wu H. Li C. Sun X. Cheng Y. Chen Z. (2023). Identification of a monoclonal antibody against porcine deltacoronavirus membrane protein. Int. J. Mol. Sci. 24. doi: 10.3390/ijms241813934 PMC10530725 37762237

[B26] Yang Y. Xu T. Wen J. Yang L. Lai S. Sun X.. (2022). Prevalence and phylogenetic analysis of porcine circovirus type 2 (PCV2) and type 3 (PCV3) in the Southwest of China during 2020-2022. Front. Vet. Sci. 9, 1042792. doi: 10.3389/fvets.2022.1042792 36504840 PMC9731358

[B27] Zhan Y. Zhang L. H. Lin Y. Cai Y. F. Zou Y. W. Hao Z. Y.. (2021). Development and preliminary testing of a probe-based duplex real-time PCR assay for the detection of African swine fever virus. Mol. Cell Probes 59, 101764. doi: 10.1016/j.mcp.2021.101764 34534618

[B28] Zhao K. Hu R. Ni J. Liang J. He X. Du Y.. (2020). Establishment of a porcine parvovirus (PPV) LAMP visual rapid detection method. J. Virol. Methods 284, 113924. doi: 10.1016/j.jviromet.2020.113924 32621958 PMC7328634

[B29] Zhao Y. Wang L. Q. Zheng H. H. Yang Y. R. Liu F. Zheng L. L.. (2020). Construction and immunogenicity of a gE/gI/TK-deleted PRV based on porcine pseudorabies virus variant. Mol. Cell Probes 53, 101605. doi: 10.1016/j.mcp.2020.101605 32464159

[B30] Zheng H. H. Fu P. F. Chen H. Y. Wang Z. Y. (2022). Pseudorabies virus: from pathogenesis to prevention strategies. Viruses 14. doi: 10.3390/v14081638 PMC9414054 36016260

[B31] Zheng S. Shi J. Wu X. Peng Z. Xin C. Zhang L.. (2018). Presence of Torque teno sus virus 1 and 2 in porcine circovirus 3-positive pigs. Transbound Emerg. Dis. 65, 327–330. doi: 10.1111/tbed.2018.65.issue-2 29285888

